# Bilateral Acute Iris Transillumination Syndrome Following Oral Moxifloxacin Overdose

**DOI:** 10.7759/cureus.47426

**Published:** 2023-10-21

**Authors:** Israel J Mendez Bermudez, Estefania Ramirez Marquez, Sofía C Ayala Rodríguez, Armando J Ruiz-Justiz, Eduardo J Rodriguez-Garcia, Monica Gonzalez, Ileana Nieves, Marino Blasini, Carmen Santos, Armando L Oliver

**Affiliations:** 1 Ophthalmology, University of Puerto Rico School of Medicine, Medical Sciences Campus, San Juan, USA

**Keywords:** bait syndrome, bait, glaucoma, uveitis, moxifloxacin, bilateral acute iris transillumination syndrome

## Abstract

We report a case of bilateral acute iris transillumination (BAIT) syndrome caused by an overdose of oral moxifloxacin in a Hispanic female patient with no previous respiratory viral infection. A 56-year-old Hispanic female with no history of ocular illness was referred to our glaucoma service to manage her microcystic edema, swelling, and refractory ocular hypertension. Her ocular and systemic symptoms, including progressively worsening bilateral ocular pain, severe photophobia, blurred vision, nausea, and vomiting, started 14 days after an accidental overdose of oral moxifloxacin. Moxifloxacin had been prescribed to treat a complicated urinary tract infection. A slit-lamp examination revealed bilateral microcystic corneal edema and transillumination in the right temporal iris, both consistent with a diagnosis of BAIT syndrome. The existing literature on BAIT syndrome is scarce, and its etiology remains unclear. This case provides clinical evidence supporting moxifloxacin toxicity as a possible cause of BAIT syndrome. We emphasize the importance of conducting extensive research to define the mechanisms involved in moxifloxacin-induced BAIT syndrome and to search for other potential etiologies of this condition.

## Introduction

Bilateral acute iris transillumination (BAIT) syndrome is a rare condition that was first described in 2004 as acute and bilateral iridocyclitis with atypical features after systemic moxifloxacin use [1]. The clinical entity can easily be misdiagnosed as anterior uveitis since patients typically present with the following symptoms: conjunctival hyperemia, photophobia, ocular pain, and blurry vision [2]. Several clinical signs, including acute bilateral pigment loss and dispersion, iris transillumination, semi-mydriasis, trabecular meshwork hyperpigmentation, and occasional elevated intraocular pressure (IOP), are characteristic of BAIT syndrome [3]. A clear etiology of the syndrome is unknown. Prior studies have reported strong associations with recent upper respiratory infections, mainly viral ones, as well as systemic antibiotic use, particularly standard-dose oral moxifloxacin [2,4]. The majority of the described cases of BAIT syndrome have been reported in Europe, with only a handful of cases having been reported in the Americas (United States of America, Brazil, and Colombia), and none having appeared in Central America or the Caribbean [4-6]. Herein, we report on a case of BAIT syndrome in a Hispanic female patient after an accidental overdose of oral moxifloxacin.

## Case presentation

A 56-year-old Hispanic female with no history of ocular illness was sent to our glaucoma service for a consultation regarding the management of microcystic edema, swelling, and refractory ocular hypertension. Her ocular and systemic symptoms, including progressively worse bilateral ocular pain, severe photophobia, blurred vision, nausea, and vomiting, began approximately 14 days after she began taking oral moxifloxacin, which had been prescribed for a complicated urinary tract infection. She misinterpreted her primary care physician’s instructions and exceeded the prescribed dose (400 mg/day, orally) of moxifloxacin by 800 mg, for a total dose of 1,200 mg/day for seven days. Additionally, she was taking dicyclomine (20 mg/day, orally) and pantoprazole (20 mg/day, orally) due to a history of irritable bowel syndrome and gastroesophageal reflux disease. Her medical history was remarkable for hypertension and diverticulitis. The patient reported no history of smoking, alcohol, or substance abuse.

**Figure 1:**
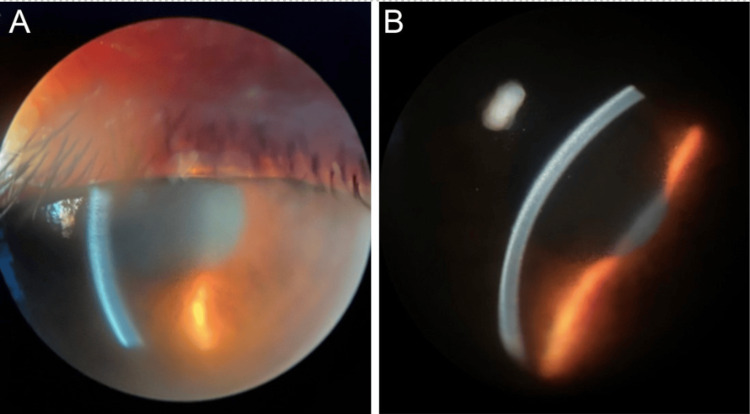
Slit-lamp examination Slit-lamp photographs of the right (A) and left (B) eyes show mid-dilated pupils (poorly responsive to light) and corneal microcystic edema.

Upon a comprehensive ophthalmic examination, her best uncorrected visual acuity was counting fingers at 6 feet in both eyes. Her IOP was 50 mmHg in the right eye and 42 mmHg in the left eye. Her pupils were mid-dilated and unreactive to light, bilaterally. Her initial slit-lamp examination revealed bilateral microcystic corneal edema and a small area of transillumination in the right temporal iris (Figure 1).

**Figure 2:**
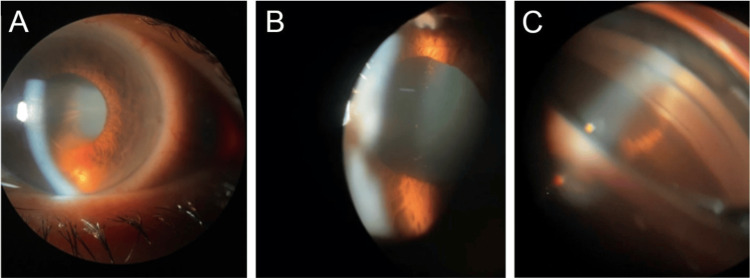
Right eye slit-lamp and gonioscopy Slit-lamp photographs of the right eye show pigment dispersion in the anterior chamber (A) and pigment deposition on the anterior lens capsule (B). A gonioscopy of the right eye shows dense inferior trabecular meshwork pigment deposition (C).

Because of her elevated IOP, her discomfort, and a poor view of the anterior chamber angle and posterior segment, medical therapy with acetazolamide (250 mg, four times daily), brimonidine 0.2%/timolol 0.5% (twice daily), and bimatoprost 0.01% (every night) was initiated. The patient’s IOP remained elevated despite topical therapy; thus, an anterior chamber tap was done with a 30-gauge needle, after which her IOP decreased to 8 mmHg and 10 mmHg in the right and left eyes, respectively. As the corneal edema cleared, a slit-lamp examination revealed bilateral pigment dispersion with 4+ pigmented cells in the anterior chamber, without flare and devoid of inflammatory cells. Gonioscopy showed dense pigment deposition in the trabecular meshwork (right eye > left eye) (Figure 2).

Her dilated funduscopic exam and optical coherence tomography were unremarkable. Laboratory tests for histocompatibility with leukocyte antigen B27 and human papillomavirus detection were negative. Her complete blood cell count and chemistry and coagulation studies were within normal limits.

**Figure 3:**
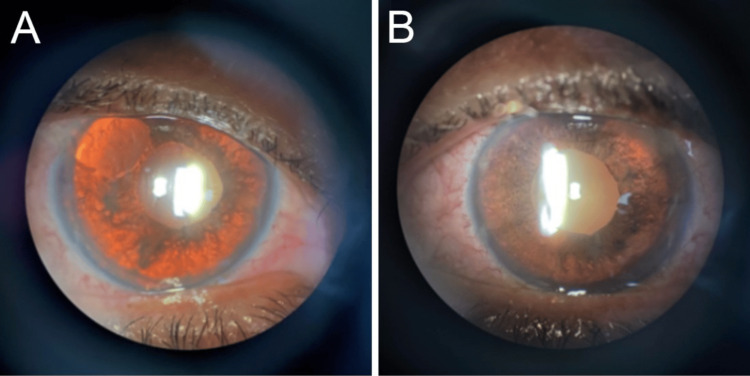
Iris transillumination defects Slit-lamp photographs of the right (A) and left (B) eyes show diffuse iris transillumination defects with semi-mydriatic distorted pupils without pharmacologic dilation.

Despite the patient having undergone an anterior chamber tap and being managed with the maximum regime of IOP-reducing medications in both eyes, her IOP increased to 24 mmHg bilaterally, and her ocular discomfort returned. She was scheduled for a right eye trabeculectomy the following week. In subsequent follow-up evaluations, iris transillumination defects were noticeably more prominent in both eyes (Figure 3).

The patient’s ocular symptoms and elevated IOP in the right eye were completely resolved after the trabeculectomy, and her uncorrected visual acuity returned to 20/25. During her following visits, the patient continued to experience decreased visual acuity (20/150) in her left eye and elevated IOP in both eyes, despite having been treated with oral acetazolamide (250 mg, 4 times daily), brimonidine 0.2%/timolol 0.5% (twice daily), and bimatoprost 0.01% (every night) and having undergone multiple anterior chamber taps. An anterior chamber washout of the left eye was done to clear excess pigment deposits in the angle in an attempt to further decrease IOP. No improvement in IOP or visual acuity was obtained after a left anterior chamber washout; hence, trabeculectomy surgery was scheduled.

After bilateral trabeculectomies, the patient’s IOP returned to the normal range (12 mmHg in the right eye and 10 mmHg in the left eye), and baseline uncorrected visual acuity was regained (20/25 in the right eye and 20/40 in the left). The patient was followed up for a month, and no glaucomatous defects were found in her optic nerve studies. Pigment dispersion in both anterior chambers decreased substantially, but the iris transillumination defects have persisted. The patient’s history and clinical findings highly suggest a diagnosis of BAIT syndrome.

## Discussion

In this report, we describe the first reported case of BAIT syndrome, in which the causative event may have been an overdose of moxifloxacin. Bilateral acute iris transillumination syndrome is characterized, as its name implies, by bilateral transillumination of the iris along with the acute onset of pigment dispersion that may occlude the trabecular meshwork, often resulting in an increase in IOP [2]. Additionally, photophobia, mydriatic pupils, and, in some cases, ocular pain have been reported [7]. Since BAIT syndrome is a rare condition, its etiology is still unclear. Based on the reports (primarily from Europe) of several different cases, the syndrome has been correlated with upper respiratory viral infections [4]. In the past decade, the debate about the etiology of BAIT syndrome has been focused on respiratory viral infections and the use of fluoroquinolones, especially moxifloxacin [4]. Moxifloxacin is a frequently used antibiotic that has not been scientifically proven to cause BAIT syndrome. However, recent articles have documented moxifloxacin intake in patients who were later diagnosed with BAIT syndrome [1, 3, 4, 6, 8].

In 2011, Tugal-Tutkun et al. concluded that BAIT syndrome might be triggered by viral infection rather than as a side effect of moxifloxacin use because only 35% of the 26 patients they described reported taking moxifloxacin [2]. However, a literature review by Perone et al. in 2019 that examined 19 articles showed that 66% of the BAIT syndrome cases analyzed in those articles involved patients who had taken moxifloxacin [4]. Based on the aforementioned finding, the authors concluded that it is impossible to entirely exclude moxifloxacin toxicity as a potential cause of BAIT syndrome.

Our case report adds a unique perspective to this debate since our patient’s moxifloxacin overdose occurred after having initiated treatment for a urinary tract infection and showed no evidence of upper respiratory viral infection. To our knowledge, this is the second reported case of BAIT syndrome in a patient with a previous bacterial urinary tract infection. In 2016, Degirmenci et al. reported on an isolated case of BAIT syndrome in a patient with an untreated *Escherichia coli *urinary tract infection, which suggests that viral infections are not the sole potential triggers of this syndrome [7].

The fact that our patient had a BAIT syndrome diagnosis following a moxifloxacin overdose (after having taken 800 mg over the prescribed daily dosage) is evidence that moxifloxacin toxicity may be a trigger for said syndrome. Previous studies have shown that the oral administration of moxifloxacin results in relatively similar concentrations in the aqueous and vitreous humor and can reach toxic concentrations in the iris pigment epithelium [4]. The proposed mechanism is that moxifloxacin has a high affinity for melanin-containing structures, such as the iris, which could result in phototoxicity [4]. In vitro studies by Perin et al. (2015) showed a decreased viability of human iris pigment cells after exposure to high concentrations of moxifloxacin [9]. Cell viability decreased by 84.12% after the administration of 100 ug/mL of moxifloxacin compared to what was seen in the control group. Though 100 ug/mL is a high concentration compared to the normal therapeutic intraocular concentration, theoretically, a moxifloxacin overdose could lead to the significant toxicity associated with the high concentration of the drug, providing a possible explanation for the onset of BAIT syndrome in our patient. Damage to ocular structures caused by a high concentration of moxifloxacin opens the possibility of considering moxifloxacin-induced BAIT syndrome as a possible diagnosis in predisposed patients who may present with increased ocular sensitivity to moxifloxacin. More research is required to investigate potential risk factors capable of increasing ocular sensitivity to moxifloxacin.

In 2021, Rivera-Valdivia et al. published a report that described a case of BAIT syndrome in a 45-year-old Colombian-Hispanic female patient who had been treated with oral moxifloxacin for acute bronchitis [8]. In this patient, the laboratory blood test was negative for infectious diseases. Additionally, her aqueous humor was analyzed by a polymerase chain reaction test, which showed her to be negative for viral entities. This case report supports our hypothesis that our syndrome might be moxifloxacin toxicity, irrespective of the presence of a respiratory viral infection.

After bilateral trabeculectomies were performed on our patient, her IOP decreased to normal ranges in both eyes, and no evidence of glaucoma was found in the follow-up visits. However, previous articles have reported that progression to glaucoma and severe sequelae are associated with BAIT syndrome [8]. Hence, clinicians should be aware of how BAIT syndrome presents clinically to prevent misdiagnosis, avoid unnecessary treatments, and limit the condition from progressing and the patient from developing other ocular complications. We propose that special attention be given to patients on moxifloxacin who present with ocular symptoms and that BAIT syndrome be considered a differential diagnosis in such cases. It is crucial to perform more extensive research to elucidate the mechanisms by which moxifloxacin might contribute to the development of BAIT syndrome.

## Conclusions

This case report presents clinical evidence supporting the theory that our patient developed BAIT syndrome as a result of moxifloxacin toxicity. In the absence of any previous viral infection in our patient, the moxifloxacin overdose emerges as the only plausible causative agent for this case of BAIT syndrome. Hence, we emphasize the importance of conducting extensive research to define the mechanisms involved in moxifloxacin-induced BAIT syndrome and search for other potential etiologies. Meanwhile, it is crucial to consider moxifloxacin toxicity as a possible trigger for the onset of BAIT syndrome in a given patient and to establish a prompt diagnosis, manage such a patient effectively, and avoid further ocular complications.
